# Optimization of Ultrasound-Assisted Pretreatment for Accelerating Rehydration of Adzuki Bean (*Vigna angularis*)

**DOI:** 10.4014/jmb.2401.01004

**Published:** 2024-02-12

**Authors:** Hyengseop Kim, Changgeun Lee, Eunghee Kim, Youngje Jo, Jiyoon Park, Choongjin Ban, Seokwon Lim

**Affiliations:** 1 Department of Food Science and Biotechnology, Gachon University, Seongnam 13120, Republic of Korea; 2 Smart Food Manufacturing Project Group, Korea Food Research Institute, Wanju 55365, Republic of Korea.; 3 Research and Development Dept., B.E.T., Busan 48119, Republic of Korea; 4 Seoul International School, Seongnam 13113, Republic of Korea; 5 Department of Environmental Horticulture, University of Seoul, Seoul 02504, Republic of Korea

**Keywords:** Adzuki bean, sonication pre-treatment, increased water absorption, optimal processing condition, response surface methodology

## Abstract

Adzuki bean (*Vigna angularis*), which provides plant-based proteins and functional substances, requires a long soaking time during processing, which limits its usefulness to industries and consumers. To improve this, ultrasonic treatment using high pressure and shear force was judged to be an appropriate pretreatment method. This study aimed to determine the optimal conditions of ultrasound treatment for the improved hydration of adzuki beans using the response surface methodology (RSM). Independent variables chosen to regulate the hydration process of the adzuki beans were the soaking time (2-14 h, X_1_), treatment intensity (150-750 W, X_2_), and treatment time (1-10 min, X_3_). Dependent variables chosen to assess the differences in the beans post-immersion were moisture content, water activity, and hardness. The optimal conditions for treatment deduced through RSM were a soaking time of 12.9 h, treatment intensity of 600 W, and treatment time of 8.65 min. In this optimal condition, the values predicted for the dependent variables were a moisture content of 58.32%, water activity of 0.9979 a_w_, and hardness of 14.63 N. Upon experimentation, the results obtained were a moisture content of 58.28 ± 0.56%, water activity of 0.9885 ± 0.0040 a_w_, and hardness of 13.01 ± 2.82 g, confirming results similar to the predicted values. Proper ultrasound treatment caused cracks in the hilum, which greatly affects the water absorption of adzuki beans, accelerating the rate of hydration. These results are expected to help determine economically efficient processing conditions for specific purposes, in addition to solving industrial problems associated with the low hydration rate of adzuki beans.

## Introduction

The adzuki bean (*Vigna angularis*) is a legume belonging to the Fabaceae family [1]. Typically cultivated and consumed in the Eastern Asia region, red beans are known to have a lower fat content and higher carbohydrate content than beans [2]. Countries in East Asia typically consume adzuki beans with rice, as porridge, or as filling in leavened or unleavened bread [3]. [4], and are beneficial for those with diabetes [5], or obesity [6, 7].

Beans, including adzuki beans are important suppliers of plant-based proteins and functional substances but require hydration for processes such as germination and fermentation [8]. In the seed coat of beans are the hilum, a scar marking the former attachment of the bean to the plant stalk, and the micropyle, a microscopic hole. The seed coat, hilum, and micropyle of beans independently correlate with the permeability of beans [9]. The hilum is very porous and is the most important bean structure that absorbs water and is a dominant factor in water inflow [10, 11]. Depending on the type, beans require 12-24 h for the complete absorption of water [12], and beans require 8-10 h to reach a 50% absorption rate [11]. This long soaking time increases preparation costs in order to achieve the desired effect [13]. Hydration limits industrial processing and requires a lot of time, thus necessitating technology that can reduce the time span.

Ultrasonic waves are pressure waves with frequencies exceeding 20 kHz. Ultrasound technology is used in the food industry for multiple purposes such as the pyrolysis of water, formation of radicals, cell destruction by inducing the structural decomposition of solvents and solutes, extraction, activation or inactivation of enzymes, mixing and homogenization, preservation, stabilization, and dissolution and crystallization [14]. As a technology that minimizes processing, ultrasound treatment can be used to preserve food quality while ensuring its safety, and depending on its frequency, it can be applied in food processing, analysis, and quality control [15]. The application of high-power ultrasonic waves in an aqueous medium forms cavitations that generate high shear force and pressure in their collapse [16]. Ghafoor *et al*. [17] have demonstrated that ultrasound treatment on navy beans (*Phaseolus vulgaris*) improved mass transfer dynamics, thus showing effective diffusivity of water transport, suggesting that ultrasound treatment could shorten the soaking time.

In the field of food engineering, ultrasound technology is commonly used. Although it has been discovered that ultrasound treatment effectively improves the hydration rate in beans, the optimal conditions of ultrasound treatment for the highest efficacy rate still need to be presented. In addition, the specific conditions demanded by industries can only be satisfied by prediction through modeling. This study has monitored the trend of the improved hydration rate of adzuki beans post-ultrasound treatment and investigated the influence of variables that affect the characteristics of adzuki beans through RSM.

Therefore, the effects of these parameters on the rehydration of adzuki beans were investigated in this study, with the comparison based on changes in the water contents, water activity (*A*_w_), hardness, and surface microstructure after each pretreatment.

## Materials and Methods

### Material

The adzuki beans (*Vigna angularis*) used in the study were provided by the Department of Southern Area Crop Science (Republic of Korea). Seeds of masses of 0.197 ± 0.02 g were used, and for maintenance purposes, they were refrigerated at 4°C. The initial moisture content and water activity were respectively 11.02 ± 0.38% and 0.5874 ± 0.0073 a_w_.

### Ultrasound Treatment

30 g of adzuki beans and 50 ml of distilled water were placed in a beaker and treated with ultrasound using an ultrasonic processor (VC750, Sonics & Materials, Inc., USA). The beans were treated under various conditions with ultrasound: at power levels of 150-750 W and at time levels of 1-10 min. During treatment, the temperature was maintained at 32 ± 2°C, and the probe of the ultrasonic processor was submerged 2 cm from the surface level. Treated beans were dried at 60°C for an hour and immersed in 150 ml of distilled water. The beans in submergence were preserved at 27°C.

### Value of Dependent Variable Determination

**Moisture content determination.** Upon extracting 3 g of adzuki beans at designated times and drying them superficially with a wiper, the moisture content was determined using a moisture analyzer (MB45, Ohaus Corporation, USA). Using the loss on drying method, the moisture analyzer calculates the loss of mass once an internal halogen dryer heats the sample at 160°C for the complete evaporation of moisture.

**Water activity determination.** Upon extracting seven adzuki beans at designated times, drying them superficially with a wiper, and crushing them with a hammer, the water activity was determined using a water activity meter (AquaLab 4TE, Meter Group Inc., USA). The water activity meter functions by the dew point measuring method in which the humidity of the sample and air in the chamber equilibrates to a temperature of 25°C and the temperature in which the steam convert to the water activity value.

**Hardness determination.** Hardness was determined using a texture analyzer (TA-XT plus, Stable Micro Systems Ltd., UK) and modifying the method [18]. The test speed was set as 1 mm/s and the samples were compressed to 90%. Used were a 2 mm cylindrical probe (P/2) and the Return-to-test method analyzing the peak point in which maximum force is applied. For determining the hardness of beans, the P/2 probe is the most commonly used for it measures both the hard seed coat and the cotyledon, which softens as it absorbs water, with its small surface [19]. 10 samples were selected at random, penetrated at the hilum by the probe, averaged, and expressed in (N) units.

### Response Surface Methodology (RSM)

The Box-Behnken Design (BBD), an RSM technique involving 3 variables, was deemed appropriate to analyze the relationship and trend of the variables by modeling the curvature of the predicted data [20, 21]. The independent variables and their ranges were set as the soaking time (2-14 h, X_1_), treatment intensity (150-750 W, X_2_), and treatment time (1-10 min, X_3_). The ranges of the three independent variables were encoded as -1, 0, and 1 (minimum value, intermediate value, maximum value), and the ultrasound treatment conditions were set in accordance with the 15-run method. The experiment was conducted with the conditions designed by BBD, and moisture content (Y_1_), water activity (Y_2_), and hardness (Y_3_) were the dependent variables measured that change depending on the independent variables. The dependent variables were expressed by the following quadratic polynomial (Eq. 1).

*Y_n_* = *β_0_*+*β_1_X_1_*+*β_2_X_2_*+*β_3_X_3_*+*β_12_X_1_X_2_*+*β_23_X_2_X_3_*+*β_13_X_1_X_3_*+*β_11_X_1_^2^*+*β_22_X_2_^2^*+*β_33_X_3_^2^* (1)

*Y_n_* is the response and *β_0_* is the intercept coefficient. Additionally, *β_1_*, *β_2_*, and *β_3_* are linear coefficients for each variable, *β_12_*, *β_23_*, and *β_13_* are coefficients for the correlation amongst each variable, and *β_11_*, *β_22_*, and *β_33_* represent secondary coefficients of each variable.

### Statistical Analysis

Values determined were averaged to be used for regression analysis: the moisture content and water activity for each treatment condition were measured 3 times, and the hardness was measured using 10 beans. Results from the experiment were used in regression analysis through the SAS program (RSREG in the Statistical Analysis System; SAS Institute Inc., USA), and the surface plot of the regression equation was presented using the Sigma plot (10 version, Systat Software Inc., Germany). The optimal conditions were predicted using Minitab (Minitab Statistical Software, Minitab Inc., USA). Optimal conditions were determined by setting the dependent variables of moisture content and water activity to the maximum and hardness to the minimum and selecting the best fit amongst the optimal conditions presented through optimization within the experimented range of soaking time, treatment intensity, and treatment time.

### Scanning Electron Microscope (SEM)

The changed hilum due to different ultrasound treatments was observed through SEM (JSM-7500F, Jeol Ltd., Japan). First, the samples were dried at 65°C for 24 h in preparation for treatment. Then, they were coated with platinum with a sputter coater (108 Auto, Ted Pella Inc., USA) while attached to carbon tape and enlarged 80 times at an acceleration voltage of 15 kV.

## Results and Discussion

### Analyzation of Optimal Conditions for Ultrasound Treatment through RSM

RSM is a mathematical and statistical optimization method used to achieve optimal conditions through the modeling and analysis of the trend influenced by multiple variables [22]. BBD is an RSM technique that determines the optimal conditions for optimal results [23].

The independent variables controlling the ultrasound treatment were the soaking time (2-14 h, X_1_), treatment intensity (150-750 W, X_2_), and treatment time (1-10 min, X_3_), set to optimize the hydration rate of the adzuki bean. Under BBD, the ranges of each variable were encoded as 3 levels, designed to have a minimum value, intermediate value, and maximum value of -1, 0, and 1, respectively, as displayed in Table 1. The dependent variables used in the regression analysis to determine the optimal ultrasound treatment conditions were moisture content (Y_1_), water activity (Y_2_), and hardness (Y_3_). Table 2 presents the 15 test conditions including the 3 intermediate values BBD designated and the dependent variables' responses. Table 3 reports the regression equation demonstrating the relationship between the independent and dependent variables, coefficient of determination R^2^, the *p*-value, and the F-value determined through the designed BBD model. Table 4 displays the different coefficients of the quadratic regression equation such as that of the linear (X_1_, X_2_, X_3_), quadratic (X_1_^2^, X_2_^2^, X_3_^2^), and cross-product (X_1_X_2_, X_1_X_3_, X_2_X_3_) of the dependent variables and their significance results.

### Moisture Content

**Fig. 1:**
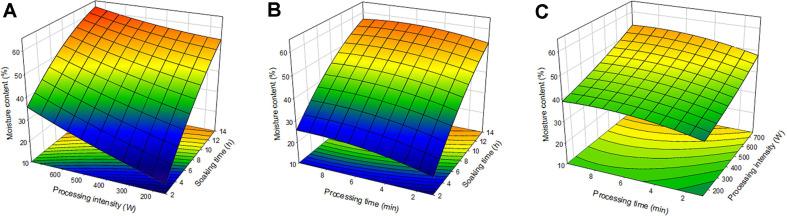
Response surface plot for moisture content of Adzuki bean. (**A**) the effect of soaking time and processing intensity, (**B**) the effect of soaking time and processing time, (**C**) the effect of processing intensity and processing time.

Moisture content affects characteristics such as conductivity to heat and electricity and density and affects the design of technical processes [24]. Since moisture exists in high quantities in food and its surrounding atmosphere, moisture content is a commonly completed analysis in the food industry. According to the analysis of variance (ANOVA), the R^2^ value of the regression equation was 0.9621, proving the model appropriate, and the *p*-value was 0.0047, proving the experimental results appropriate. In Table 3, the coefficients of the regression equation of Y_1_ are in descending order of soaking time X_1_ (5.548426), treatment intensity X_3_ (1.986502), and treatment time X_2_ (0.026775), demonstrating that soaking time, treatment intensity, and treatment time, respectively, had the greatest effect on the moisture content. Only for the value of ‘X_1_’ was the regression coefficient of the regression equation of moisture content statistically significant (Table 4). Fig. 1 shows the 3-dimensional graph produced by the regression equation. Fig. 1A displays the effect of soaking time and treatment intensity on the moisture content of the adzuki bean, and the moisture content increased as the soaking time and treatment intensity increased, with the highest moisture content of 62.18% derived at the treatment condition of 14 h and 750 W. The effect of soaking time and treatment time was described in Fig. 1B, and similarly to the graph of 1A, the moisture content increased as the time submerges increased, the highest value of 57.59% derived at 14 h and 7 min. Fig. 1C shows the effect of treatment intensity and treatment time, and the moisture content increased as the treatment intensity and treatment time increased, the maximal moisture content of 53.72% being derived at 750 W and 9 min. Generally, high values of soaking time, treatment intensity, and treatment time caused an increase in moisture content. The study by Ulloa [25] demonstrated that as the treatment time increased, the moisture saturation time of various beans decreased, the soaking time decreased by approximately 34%. Moisture content is affected the most by the soaking time and judging by the effect of treatment intensity and treatment time, ultrasound treatment is suggested to have an auxiliary role in the moisture content of adzuki beans.

### Water Activity

**Fig. 2:**
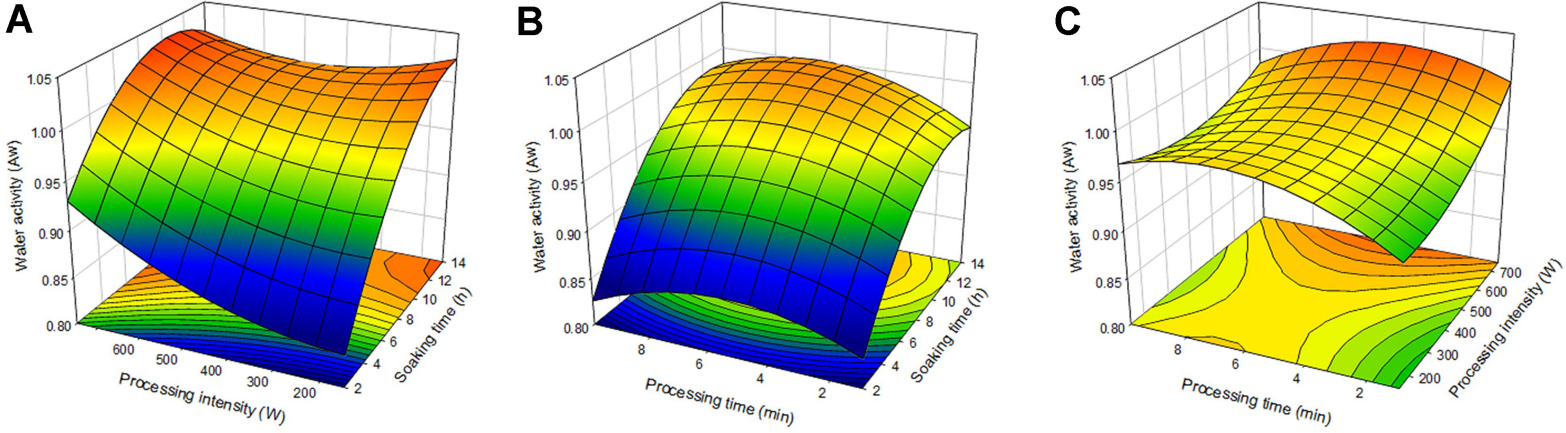
Response surface plot for water activity of Adzuki bean. (**A**) the effect of soaking time and processing intensity, (**B**) the effect of soaking time and processing time, (**C**) the effect of processing intensity and processing time.

Typically water activity differs depending on the characteristics of the product such as its structure, effect of solvents, and surface activity [26]. Change in water activity is one of the leading causes of change in food quality and is a commonly completed analysis with moisture content. According to the results of ANOVA, the R^2^ value of the total model of the regression equation was 0.8875 and the *p*-value 0.0591, thus not significant. Also, the regression equation is as follows, but the *p*-value of lack-of-fit is 0.0260 which is not appropriate. In Table 3, the coefficients of the regression equation of Y_2_ are in descending order of soaking time X_1_ (0.038427), treatment time X_3_ (0.021156), and treatment intensity X_2_ (0.000034426), demonstrating that soaking time, treatment time, and treatment intensity, respectively, had the most significant effect on the water activity. The regression coefficient of the regression equation of water activity was statistically significant for the values of the ‘Intercept’ and ‘X_1_’ (Table 4). Fig. 2 displays the 3-dimensional graph produced by the regression equation. Fig. 2A displays the effect of soaking time and treatment intensity on the water activity of the adzuki bean, and generally, as the soaking time increased the water activity increased. At 2 h of submergence, as the treatment intensity increased, the water activity increased from 0.8333 a_w_ (150 W) to 0.9299 a_w_ (750 W), but at 14 h of submergence, the water activity decreased from 1.0242 a_w_ (150 W) to 0.9995 a_w_ (450 W) and it increased back up to 1.0181 a_w_ (750 W) after 450 W. Beyond 11 h of submergence, the water activity values of nearly all treatment intensities were above 1 a_w_, and as these values were predicted through RSM, they are analyzed to have water activity values close to 1 a_w_. The effect of soaking time and treatment time was described in Fig. 2B, and in general, as the soaking time increased the water activity increased. At 2 h of submergence, as the treatment time increased, the water activity increased from 0.8305 a_w_ (1 min) to 0.8600 a_w_ (5.5 min), but after 5.5 min of treatment, the water activity decreased to 0.8257 a_w_ (10 min). Fig. 2C displays the effect of treatment intensity and treatment time, and at a treatment intensity of 750 W, as the treatment time increased, the water activity increased from 1.0007 a_w_ (1 min) to 1.0243 a_w_ (4.6 min), but after 4.6 min of treatment, it decreased to 0.9830 a_w_ (10 min). Generally, the water activity increased as the soaking time increased, similarly to the moisture content. The water activity increased then decreased as the treatment time increased. Mothibe *et al*. [27] treated apples with various treatment times and once the apples were dried and water activity determined, there was a higher water activity rate in the apple treated for 5 min than the one treated for 15 min, and it was demonstrated that as the treatment time increases, more soluble solids are lost. In the 3-dimensional graph, some values of the water activity are larger than 1 a_w_, which appears to be due to RSM predicting the values of dependent variables using multiple variables.

### Hardness

**Fig. 3:**
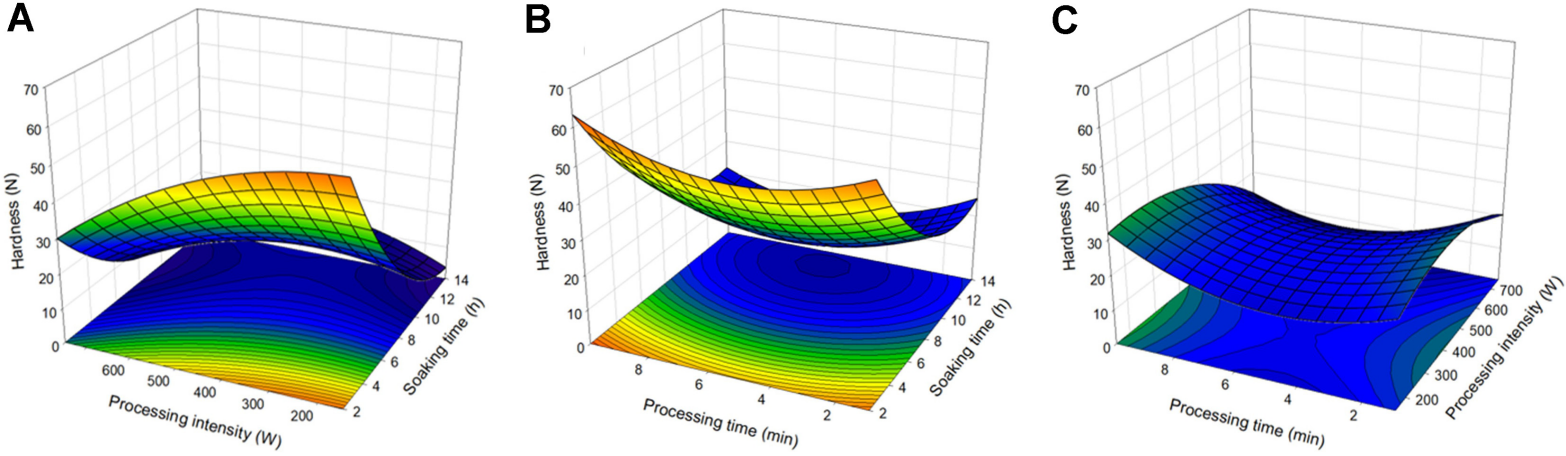
Response surface plot for hardness of Adzuki bean. (**A**) the effect of soaking time and processing intensity, (**B**) the effect of soaking time and processing time, (**C**) the effect of processing intensity and processing time.

The hydration of the adzuki bean is an effective method to reduce its hard texture. The hardness of a bean is an important evaluator of its physical characteristics. According to ANOVA, the R^2^ value of the total model of the regression equation was 0.9019, showing high explanatory power, and the *p*-value was 0.0437, showing statistical significance. Also, the *p*-value of lack-of-fit is 0.0111, so it is not an appropriate model, but the regression equation is as follows. In Table 3, the coefficients of the regression equation of Y_3_ are in ascending order of soaking time X_1_ (-10.723843), treatment time X_3_ (-3.265617), and treatment intensity X_2_ (0.026619), demonstrating that X_1_, X_3_, X_2_ respectively, had the greatest effect on the water activity. Beans soften as they absorb water, thus the lower the coefficients of the independent variables, the larger the effect they have on the hardness. The regression coefficient of the regression equation of durability was statistically significant for the values of the ‘Intercept’ and ‘X_1_’ (Table 4). The regression equation was used to display a 3-dimensional graph in Fig. 3. Fig. 3A displays the effect of soaking time and treatment intensity on the hardness of the adzuki bean. At a treatment intensity of 150 W, as the soaking time increased, the hardness decreased from 60.28 N (2 h) to 3.48 N (14 h), at 750 W, the hardness decreases from 30.18 N (2 h) to 5.72 N (10.4 h), and at 450 W, it increases to 10.37 N (14 h). The effect of soaking time and treatment time is described in Fig. 1B, and as the soaking time increased the durability decreased, and as the treatment time increased, the durability decreased from 30.04 N (8 h, 1 min) to 20.78 N (8 h, 5.5 min), and after 5.5 min, it increased to 29.77 N (8 h, 10 min). Fig. 3C shows the effect of treatment intensity and treatment time, and as the treatment intensity increased the durability increased from 19.27 N (150 W, 5.5 min) to 21.93 N (330 W, 5.5 min) then decreased to 7.67 N (750 W, 5.5 min). Also, as the treatment time increased, the durability decreased from 30.04 N (450 W, 1 min) to 20.78 N (450 W, 5.5 min) and increased back to 29.77 N (450 W, 10 min). Generally, the durability was highly affected by the treatment time, and as the soaking time increased, the durability decreased then increased. Li *et al*. [28] demonstrated that as the soaking time was prolonged, the durability of the sample increased as water bonding with macromolecules such as proteins and starch increased.

### Optimum Condition

Based on each dependent variablés regression equation and graph, the optimal treatment conditions and their predicted values of dependent variables are represented in Table 5. The optimal ultrasound treatment conditions were an immersion time of 12.9 h, treatment intensity of 600 W, and treatment time of 8.65 min when maximizing the moisture content and water activity while minimizing the hardness. The values predicted for the dependent variables were a moisture content of 58.32%, water activity of 0.9979 a_w_, and hardness of 14.63 N. When experimenting with the optimal conditions determined by RSM, the results obtained were a moisture content of 58.28 ± 0.56%, water activity of 0.9885 ± 0.0040 a_w_, and hardness of 13.01 ± 2.82 N, the experimented values confirming a 95% confidence interval except for the water activity. Values of water activity are accepted as accurate within an error range of 0.01 a_w_ in the food industry to determine if the value has reached a critical zone in which decomposition reaction can occur [26].

### Scanning Electron Microscopy of the Bean Hilum

**Fig. 4:**
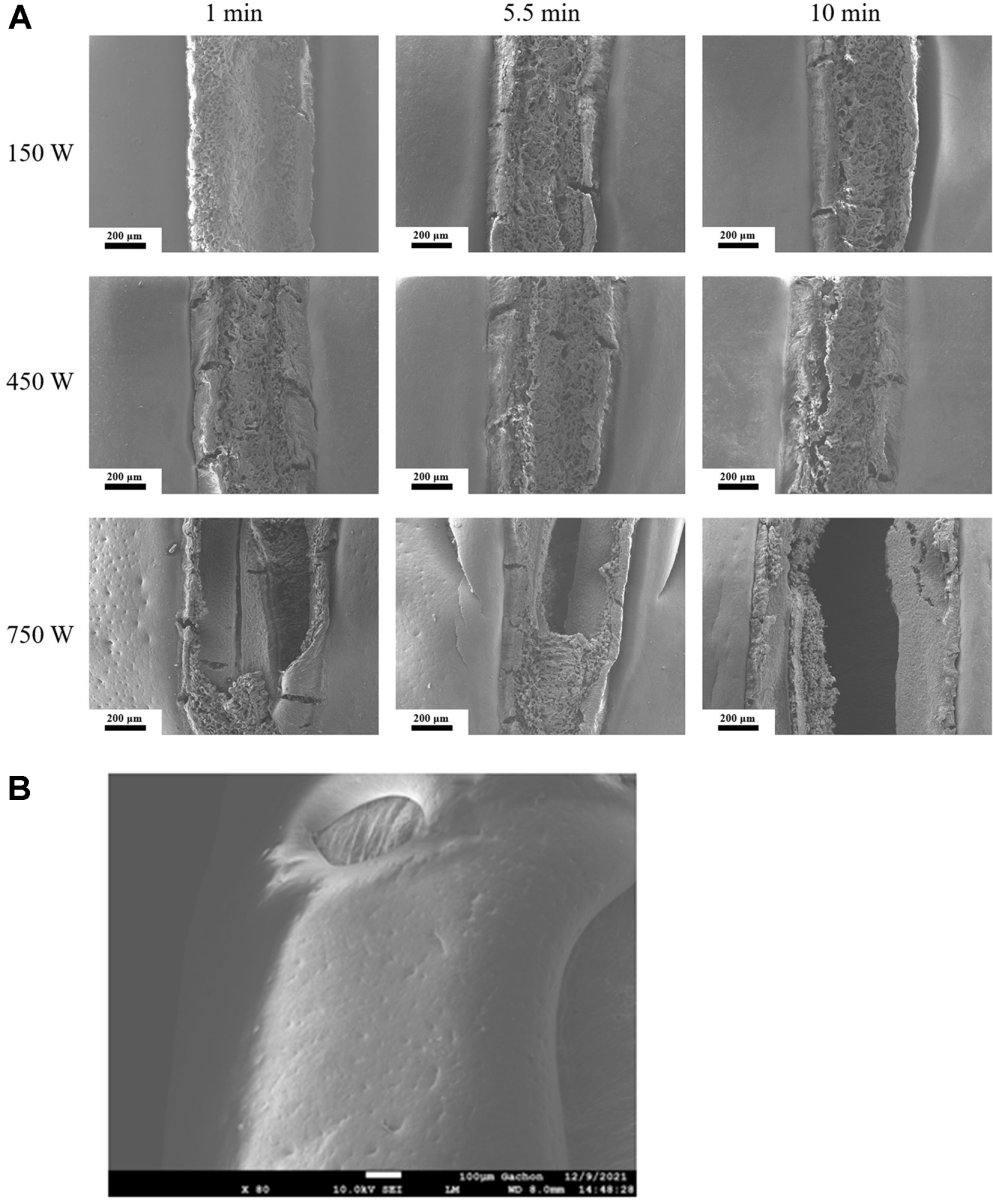
Scanning electron micrographs of Adzuki beans sonicated various conditions (X80). (**A**) Scanning electron micrographs of Adzuki beans hilum sonicated at different levels of amplitude and time, (**B**) Surface of red beans sonicated at 750 W for 10 min.

The seed coat of the adzuki bean is highly impermeable, hence the hilum has a large effect on the hydration of the beans [29]. The changes in the hilum were observed through SEM, and Fig. 4A displays the conditions of the hilum that have undergone various ultrasound treatments. Depending on the treatment, abrasions and crevices of the hilum were exacerbated, and the prolonged treatment time produced a rough hilum due to the larger scale abrasions (450 W, 5.5 min) and crevices formed in the later stage of treatment in the central part of the hilum (450 W, 10 min). Changes to the hilum due to the treatment time were equally damaging in treatment intensities of 150 W and 750 W, but the treatment at 150 W did not produce crevices in the hilum whereas the treatment at 750 W did within a minute of treatment. The adzuki bean treated at 750 W for 10 min showed damage in areas other than the hilum as it produced a large hole in its surface (Fig. 4B). Through these observations, it was demonstrated that the surface layer of the hilum formed abrasions and crevices due to the strong shear force of the ultrasound treatment, and the increase in treatment intensity and time gradually intensified the damage on the surface.

## Conclusion

This study analyzed the optimal conditions for an increase in the hydration rate of adzuki beans. The sonication process helped in the diffusion of moisture in Adzuki beans. All dependent variables were highly affected by the independent variables in the order of soaking time, treatment time, and treatment intensity. The optimal conditions for treatment analyzed through RSM were an immersion time of 12.9 h, treatment intensity of 600 W, and treatment time of 8.65 min, and the experimented values proved similar to the predicted values. Appropriate ultrasound treatment increased the rate of hydration by producing abrasions and crevices in the hilum through which the bean absorbs water, but extreme treatment damaged the surface of the bean as well by creating large crevices. The optimal conditions for ultrasound treatment were determined in this study for the treatment of red beans, and the predictive model presented by the study is expected to provide specific treatment conditions required by industries in addition to the optimal ones.

## Figures and Tables

**Table 1:** Level of independent variables for experimental design.

Factor	Independent variable	Levels		
		-1	0	1
X_1_	Soaking time (h)	2	8	14
X_2_	Processing intensity (W)	150	450	750
X_3_	Processing time (min)	1	5.5	10

**Table 2:** Box-behnken design matrices and responses for experimental values.

Run	X_1_	X_2_	X_3_	Y_1_	Y_2_	Y_3_
	Soaking time (h)	Processing intensity (W)	Processing time (min)	Moisture content (%)	Water activity (a_w_)	Hardness (N)
1	2	150	5.5	18.42	0.8576	55.56
2	14	150	5.5	54.98	0.9949	13.73
3	2	750	5.5	35.29	0.9589	19.92
4	14	750	5.5	58.32	0.9936	15.10
5	2	450	1	18.54	0.8193	66.74
6	14	450	1	57.51	0.9952	17.19
7	2	450	10	21.70	0.7832	71.38
8	14	450	10	56.35	0.9933	14.55
9	8	150	1	29.60	0.9123	22.86
10	8	750	1	47.65	0.9825	23.76
11	8	150	10	38.87	0.9857	28.40
12	8	750	10	57.82	0.9960	15.35
13	8	450	5.5	45.45	0.9821	19.28
14	8	450	5.5	41.61	0.9702	21.06
15	8	450	5.5	46.37	0.9861	22.00

**Table 3:** Polynomial equation calculated by RSM program for ultrasonic treatment conditions of red bean.

Response	Quadratic polynomial model	R^2^	*F*-value	*p*-value	Lack-of-fit
Moisture content	Y_1_= -7.306341 +5.548426X_1_ +0.026775X_2_ +1.986502X_3_ -0.106725X_1_^2^ -0.001879X_2_X_1_ +0.000012421X_2_^2^ -0.040000X_3_X_1_ +0.000167X_3_X_2_ –0.104177X_3_^2^	0.9621	14.12	0.0047	0.1678
Water activity	Y_2_= 0.692674 +0.038427X_1_ +0.000034426X_2_ +0.021156X_3_ -0.001383X_1_^2^ -0.000014250X_2_X_1_ +0.000000240X_2_^2^ +0.000317X_3_X_1_ -0.000011093X_3_X_2_ -0.001576X_3_^2^	0.8875	4.38	0.0591	0.0260
Hardness	Y_3_= 83.837276 –10.723843X_1_ +0.026619X_2_ –3.265617X_3_ +0.349583X_1_^2^ +0.005140X_2_X_1_ –0.000080972X_2_^2^ –0.067407X_3_X_1_ –0.002583X_3_X_2_ +0.449383X_3_^2^	0.9019	5.11	0.0437	0.0111

**Table 4:** Estimated coefficients of quadratic polynomial equation for different response.

	Y_1_		Y_2_		Y_3_	
	Coefficient	P-value	Coefficient	P-value	Coefficient	P-value
Intercept	-7.306341	0.5156	0.692674	0.0005	83.837276	0.0137
X_1_	5.548426	0.0094	0.038427	0.0200	-10.723843	0.0144
X_2_	0.026775	0.3956	0.000034426	0.8929	0.026619	0.6862
X_3_	1.986502	0.3037	0.021156	0.2073	-3.265617	0.4221
X_1_*X_1_	-0.106725	0.1773	-0.001383	0.0606	0.349583	0.0628
X_2_*X_1_	-0.001879	0.2099	-0.000014250	0.2522	0.005140	0.1275
X_2_*X_2_	0.000012421	0.6670	0.000000240	0.3435	-0.000080972	0.2257
X_3_*X_1_	-0.040000	0.6653	0.000317	0.6842	-0.067407	0.7342
X_3_*X_2_	0.000167	0.9275	-0.000011093	0.4840	-0.002583	0.5221
X_3_*X_3_	-0.104177	0.4282	-0.001576	0.1825	0.449383	0.1452

**Table 5:** Comparison between predicted and observed values of response variables within the range of the optimum condition.

Response	Predicted condition	Predicted value	Experimental value
Moisture content	12.9 h, 600 W, 8.65 min	58.32%	58.28±0.56%
Water activity		0.9979 a_w_	0.9885±0.0040 a_w_
Hardness		14.63 N	13.01±2.82 N
